# Topical Bixin Confers NRF2-Dependent Protection Against Photodamage and Hair Graying in Mouse Skin

**DOI:** 10.3389/fphar.2018.00287

**Published:** 2018-03-27

**Authors:** Montserrat Rojo de la Vega, Donna D. Zhang, Georg T. Wondrak

**Affiliations:** ^1^Department of Pharmacology and Toxicology, College of Pharmacy, University of Arizona, Tucson, AZ, United States; ^2^The University of Arizona Cancer Center, University of Arizona, Tucson, AZ, United States

**Keywords:** NRF2, bixin, UV, PUVA, skin oxidative stress, photodamage, sunburn, hair graying

## Abstract

Environmental exposure to solar ultraviolet (UV) radiation causes acute photodamage, premature aging, and skin cancer, attributable to UV-induced genotoxic, oxidative, and inflammatory stress. The transcription factor NRF2 [nuclear factor erythroid 2 (E2)-related factor 2] is the master regulator of the cellular antioxidant response protecting skin against various environmental stressors including UV radiation and electrophilic pollutants. NRF2 in epidermal keratinocytes can be activated using natural chemopreventive compounds such as the apocarotenoid bixin, an FDA-approved food additive and cosmetic ingredient from the seeds of the achiote tree (*Bixa orellana*). Here, we tested the feasibility of topical use of bixin for NRF2-dependent skin photoprotection in two genetically modified mouse models [SKH1 and C57BL/6J (*Nrf2^+/+^* versus *Nrf2^-/-^*)]. First, we observed that a bixin formulation optimized for topical NRF2 activation suppresses acute UV-induced photodamage in *Nrf2^+/+^* but not *Nrf2^-/-^* SKH1 mice, a photoprotective effect indicated by reduced epidermal hyperproliferation and oxidative DNA damage. Secondly, it was demonstrated that topical bixin suppresses PUVA (psoralen + UVA)-induced hair graying in *Nrf2^+/+^* but not *Nrf2^-/-^* C57BL/6J mice. Collectively, this research provides the first *in vivo* evidence that topical application of bixin can protect against UV-induced photodamage and PUVA-induced loss of hair pigmentation through NRF2 activation. Topical NRF2 activation using bixin may represent a novel strategy for human skin photoprotection, potentially complementing conventional sunscreen-based approaches.

## Introduction

Environmental exposure to solar ultraviolet (UV) radiation causes acute photodamage, premature aging, and skin cancer, all of which may originate from UV-induced genotoxic, oxidative, neuroendocrine, and inflammatory stress (Wondrak et al., 2006; Slominski, 2007; Chen et al., 2014; Natarajan et al., 2014; Panich et al., 2016; Rojo de la Vega et al., 2017; Lin et al., 2018). In response to environmental stressors, the redox-sensitive transcription factor NRF2 [nuclear factor erythroid 2 (E2)-related factor 2] orchestrates major cellular defense mechanisms including phase II detoxification, inflammatory signaling, DNA repair, antioxidant response, and autophagy activation, all of which might be involved in the maintenance of skin barrier function (Braun et al., 2002; Kerns et al., 2010; Schafer et al., 2012; Tebay et al., 2015). NRF2 is expressed in all skin cell types (Schafer and Werner, 2015), but its levels are typically low in the absence of oxidative or electrophilic stress as a result of its interaction with kelch ECH-associated protein 1 (KEAP1), a substrate adaptor for an E3 ubiquitin ligase complex that constantly ubiquitylates NRF2, promoting its proteasomal degradation (Zhang et al., 2004). Oxidative or electrophilic insults cause a conformational change in KEAP1 that prevents NRF2 ubiquitylation (Dinkova-Kostova et al., 2002; Zhang and Hannink, 2003; Baird et al., 2013), causing the accumulation of newly synthesized NRF2 that translocates to the nucleus to activate the expression of target genes containing an antioxidant response element (ARE) in their regulatory regions (Itoh et al., 1997). Numerous studies strongly suggest a protective role of NRF2-mediated gene expression against cutaneous photodamage induced by solar UV radiation as evidenced by suppression of UV-induced apoptosis and inflammatory signaling (Hirota et al., 2005; Dinkova-Kostova et al., 2006; Wondrak et al., 2008; Schafer et al., 2010; Saw et al., 2011), and research performed in SKH1 mice documents that genetic NRF2 activation protects against acute photodamage and photocarcinogenesis (Knatko et al., 2015, 2016). Our own studies have demonstrated the photoprotective effects of pharmacological NRF2 activation in cultured human skin cells and reconstructed epidermal skin models (Wondrak et al., 2008; Tao et al., 2013, 2015). In addition, based on the role of NRF2 in the control of melanocyte responses to environmental stressors, NRF2 has been implicated in cutaneous pigmentation disorders associated with redox alterations relevant to vitiligo and stress-induced hair graying (Marrot et al., 2008; Jian et al., 2014; Jadkauskaite et al., 2017). Consequently, pharmacological modulation of NRF2 has now attracted considerable attention as a novel approach to skin photoprotection, cancer photochemoprevention, and suppression of radiation dermatitis and stress-induced hair graying (Kalra et al., 2012; Tao et al., 2013; Chun et al., 2014; Reisman et al., 2014; Nakagami and Masuda, 2016).

Recently, we have demonstrated the feasibility of NRF2-dependent systemic photoprotection by dietary constituents focusing on the apocarotenoid bixin (Tao et al., 2015), an FDA-approved natural food colorant from the seeds of the achiote tree (*Bixa orellana*) native to tropical America (Ulbricht et al., 2012; Stohs, 2014). Our research revealed that systemic administration of bixin activates epidermal NRF2 with potent protective effects against acute solar UV-induced skin damage in SKH1 mice (Tao et al., 2015). Importantly, in addition to NRF2-dependent indirect antioxidant cellular effects, bixin displays molecular activities as free radical scavenger and excited state quencher (Di Mascio et al., 1990), PPAR (peroxisome proliferator-activated receptor) α/γ agonist (Takahashi et al., 2009; Goto et al., 2012), and TLR (Toll-like receptor) 4/NFκB (nuclear factor kappa-light-chain-enhancer of activated B cells) antagonist (Xu and Kong, 2017), potentially contributing to protection of skin barrier function against environmental stress.

In this follow-up study, we have for the first time explored the feasibility of NRF2-dependent skin photoprotection employing topical bixin in two genetically modified mouse models [SKH1 and C57BL/6J (*Nrf2^+/+^* versus *Nrf2^-/-^*)] in order to distinguish between NRF2-dependent and -independent photoprotective effects. First, we observed that a bixin formulation optimized for topical NRF2 activation suppresses acute UV-induced photodamage in *Nrf2^+/+^* but not *Nrf2^-/-^* SKH1 mice. Likewise, topical bixin can suppress stress-induced hair graying in an established PUVA (psoralen + UVA) regimen in *Nrf2^+/+^* but not *Nrf2^-/-^* C57BL/6J mice, confirming the NRF2-dependence of photoprotection achievable by topical application of this ethno-pharmacological skin protectant used throughout the Americas since ancient times.

## Materials and Methods

### Chemicals and Antibodies

Analytical grade *cis*-bixin (9-*cis*-6,6′-diapo-ψ,ψ-carotenedioic acid, 6-methyl ester) was purchased from Spectrum (CAS number: 6983-79-5). LC/MS confirmation of purity (>98% by weight) was performed using electrospray mass spectrometry of bixin [dissolved in tetrahydrofuran and diluted 10-fold in acetonitrile/NH_4_OH (0.1 N); ESI-MS (negative ion mode) *m/z* 393.21 (M - 1)^-^] employing a Bruker Apex FT/ICR mass spectrometer, as specified before (Tao et al., 2015). Polyethylene glycol 400 (PEG400) was from EMD Millipore. Xanthotoxin (8-methoxypsoralen, 8-MOP), and hydrogen peroxide (H_2_O_2_) were from Sigma. Primary antibodies against NRF2, KEAP1, TRXR1, GCLM, NQO1, HO1, OGG1, GAPDH, and actin were purchased from Santa Cruz Biotechnology. Primary antibody against p62 was from Abnova. Secondary antibodies conjugated with horseradish peroxidase (HRP) were purchased from Sigma. Antibody against 8-oxo-deoxyguanosine (8-oxo-dG) was from Trevigen.

### Cell Culture

Primary human epidermal melanocytes, neonatal, low pigmented (HEMnLP) were cultivated in medium 254 supplemented with 0.08 mM CaCl_2_, 1× human melanocyte growth supplement (HMGS), and 1× gentamicin/amphotericin, all purchased from Gibco. Cells were maintained in a humidified incubator with 5% CO_2_ at 37°C.

### UV Irradiation

Mice were UV irradiated utilizing a KW large area solar simulator, model 91293, from Oriel Corporation, equipped with a 1000 W Xenon arc lamp power supply, model 68920 (Wondrak et al., 2003; Williams et al., 2014; Park et al., 2015; Tao et al., 2015). The output was quantified using a dosimeter from International Light Inc. For simulated solar UV irradiation (“UV,” consisting of UVA + UVB), a VIS-IR band pass-blocking filter combined with an atmospheric attenuation filter (output 290–400 nm plus residual 650–800 nm) was used. At 345 mm from the source, the UV dose was 4.4 J/cm^2^ UVA + 240 mJ/cm^2^ UVB. For UVA irradiation (“UVA”), a VIS-IR band pass-blocking filter combined with a UVB/C blocking filter (output 320–400 nm plus residual 650–800 nm) was used. At 345 mm from the source, the UVA dose was 5.39 mJ/cm^2^ with a residual UVB dose of 3.16 μJ/cm^2^. Total dose of UVA for PUVA model was 1.32 and 0.33 J/cm^2^ for cells.

### Mouse Models

This study was carried out in accordance with the recommendations of the Guide for the Care and Use of Laboratory Animals. All protocols were approved by the University of Arizona Institutional Animal Care and Use Committee. For the experiments, mice between 8 and 12 weeks old were used. *Pilot project*: SKH1 *Nrf2^+/+^* mice (*n* = 4) were applied PEG400 (vehicle control) on one half of the dorsal skin and 1% bixin in PEG400 (w/w) on the other half. Skin tissues were collected 24 h later. *Acute photodamage*: SKH1 *Nrf2^+/+^* and *Nrf2^-/-^* mice (*n* = 6/group) were randomly allocated to one of the treatment groups: control (Ctrl: topical PEG400, no UV), UV (UV: topical PEG400, UV), bixin (Bix: 1% bixin in PEG400, no UV), bixin and UV (Bix + UV: 1% bixin in PEG400, UV). *PUVA-induced hair graying*: C57BL/6J *Nrf2^+/+^* and *Nrf2^-/-^* mice were depilated using Nair cream for ∼1 min. Three days after depilation, mice (*n* = 6/group) were randomly allocated to one of the treatment groups: control (Ctrl: topical PEG400, no UVA), bixin (Bix: 1% bixin in PEG400, no UVA), photosensitizer and UVA (PUVA: PEG400, 50 μL 0.5 mg/mL 8-MOP, UVA), bixin and PUVA (Bix + PUVA: 1% bixin in PEG400, 50 μL 0.5 mg/mL 8-MOP, UVA). See **Figures 2**, **4**, **5** for more detailed experimental time points.

### Immunoblotting

Cell lysates were collected in 1× sample buffer [50 mM Tris–HCl pH 6.8, 2% sodium dodecyl sulfate (SDS), 10% glycerol, 100 mM dithiothreitol (DTT), and 0.1% bromophenol blue]. Snap-frozen skin tissue sections were homogenized in 2× sample buffer without bromophenol blue. Samples were boiled, sonicated, resolved by SDS-PAGE, and subjected to immunoblot analyses with the indicated antibodies. Images were obtained with an Azure c600 chemiluminescence imager.

### Histological Analyses

Skin tissues were fixed in 10% buffered formalin and embedded in paraffin. Sections (5 μm) were baked and deparaffinized for histological staining. Hematoxylin and eosin (H&E) staining was performed for pathological examination. For immunohistochemical analyses, antigen retrieval was done by boiling the tissue slides in citrate buffer (pH 6.0) or EDTA buffer (pH 8.0). Endogenous peroxidase was blocked with 10% hydrogen peroxide and 5% normal goat serum was used for blocking. Tissue sections were incubated overnight with the indicated antibodies and staining was performed using the EnVision+System-HRP (DAB) kit from Dako, according to the manufacturer’s instructions. For detection of oxidative DNA damage, 8-oxo-dG staining was performed according to the manufacturer’s instructions (Trevigen). Images were obtained with a Nikon Eclipse 50i microscope and the Nikon NIS Elements F 4.0 software.

### Image Analyses and Quantification

Immunoblot, immunohistochemistry, and whole mouse images were analyzed using ImageJ. Briefly, band intensities and staining intensities were measured for equal areas as mean pixel intensity. Results were normalized and expressed as fold change compared to controls.

### Statistical Analyses

Results are presented as means ± SEM. Paired Student’s *t*-tests were used to compare the means of groups. A *p* < 0.05 was deemed significant. Experiments were performed in triplicate unless otherwise indicated.

## Results

### Topical Bixin Activates NRF2 in SKH1 Mouse Skin

**FIGURE 1:**
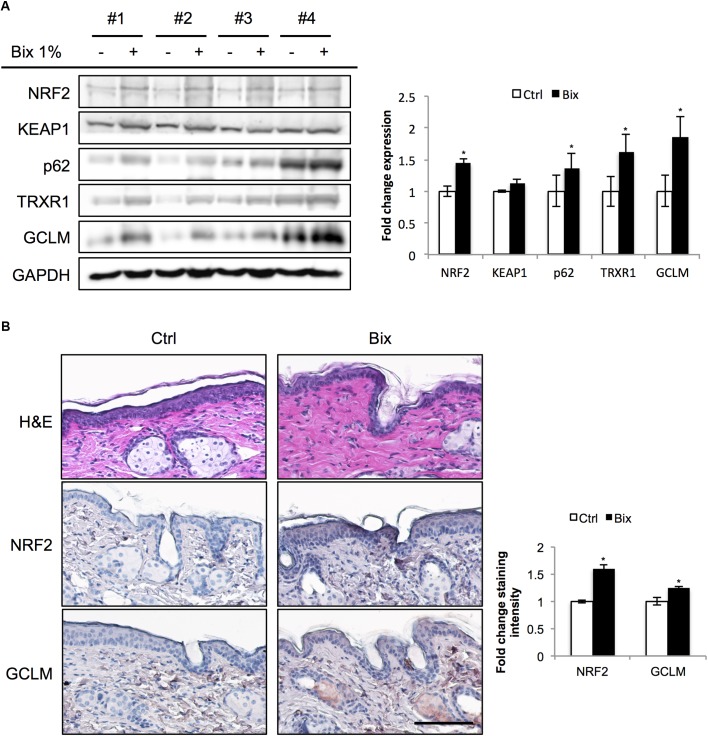
Topical bixin application activates NRF2 in SKH1 mouse skin. SKH1 *Nrf2^+/+^* mice were treated with PEG400 vehicle control (-) on half of their dorsal skin and 1% bixin (+) on the other half. Skin tissues were collected 1 day later. **(A)** Protein expression of NRF2 and its downstream genes in skin tissue lysates as assessed by immunoblot analyses in four individual mice. Protein expression was quantified, normalized, and represented as fold change compared to controls. **(B)** Histological analyses by H&E and immunohistochemical staining for NRF2 and its target gene GCLM. Images representative of specific treatment groups are shown. Scale bar: 10 μm. ^∗^*p* < 0.05 compared to Ctrl.

In preparation of murine photoprotection experiments, we first conducted a pilot study to test the feasibility of epidermal NRF2 activation using a topical bixin formulation (**Figure 1**). To this end, SKH1 hairless mice were treated with PEG400 vehicle control (Ctrl) on half of their dorsal skin or 1% (w/w) bixin (Bix) on the other half. Skin tissues were then collected 24 h after a single application to assess NRF2 activation. As shown by immunoblot analysis, bixin treatment upregulates NRF2 levels (1.5-fold average induction) with induction of NRF2 target gene expression (p62, TRXR1, GCLM; 1.9-fold maximum induction), an effect that occurs without modulation of KEAP1 protein levels (**Figure 1A**). Similar changes were observed by immunohistochemical analyses, indicating epidermal upregulation of NRF2 and GCLM (**Figure 1B**). Taken together, these results indicate that a topical bixin formulation efficiently activates NRF2 in SKH1 mouse skin.

### Topical Bixin Confers Protection Against UV-Induced Skin Damage in *Nrf2^+/+^* But Not in *Nrf2^-/-^* SKH1 Hairless Mice

**FIGURE 2:**
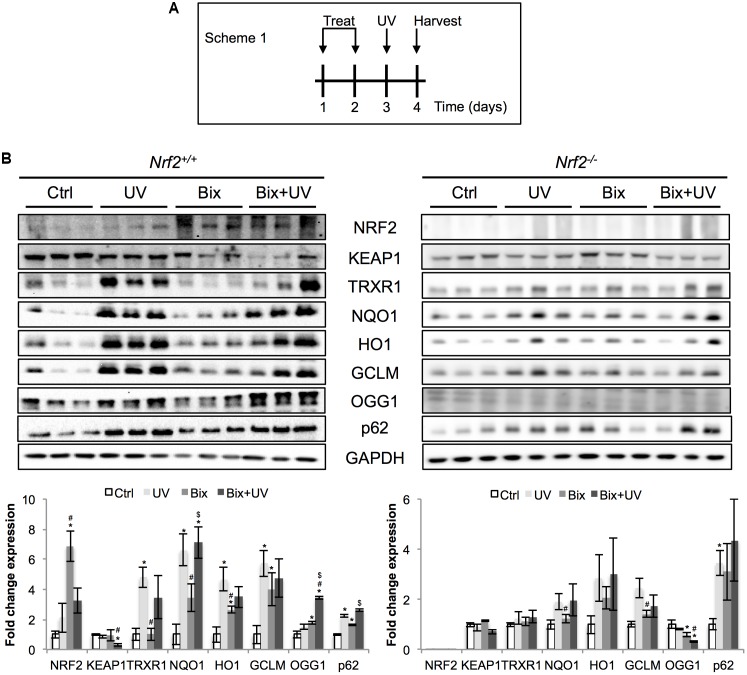
Topical bixin and UV modulate the expression of NRF2 pathway proteins in SKH1 mouse skin. **(A)** Treatment scheme of acute photodamage study. SKH1 *Nrf2^+/+^* and *Nrf2^-/-^* mice were treated with vehicle control (Ctrl) or 1% bixin (Bix), alone or in combination with solar UV (UVB 240 mJ/cm^2^) (UV, Bix + UV). Skin tissues were collected for analysis 1 day after exposures. **(B)** Protein expression of NRF2 and its downstream genes in skin tissue lysates as assessed by immunoblot analyses. Protein expression was quantified, normalized, and represented as fold change compared to controls. Blots of three representative samples for each group are shown. *p* < 0.05: ^∗^compared to Ctrl, ^#^compared to UV, ^$^compared to Bix.

**FIGURE 3:**
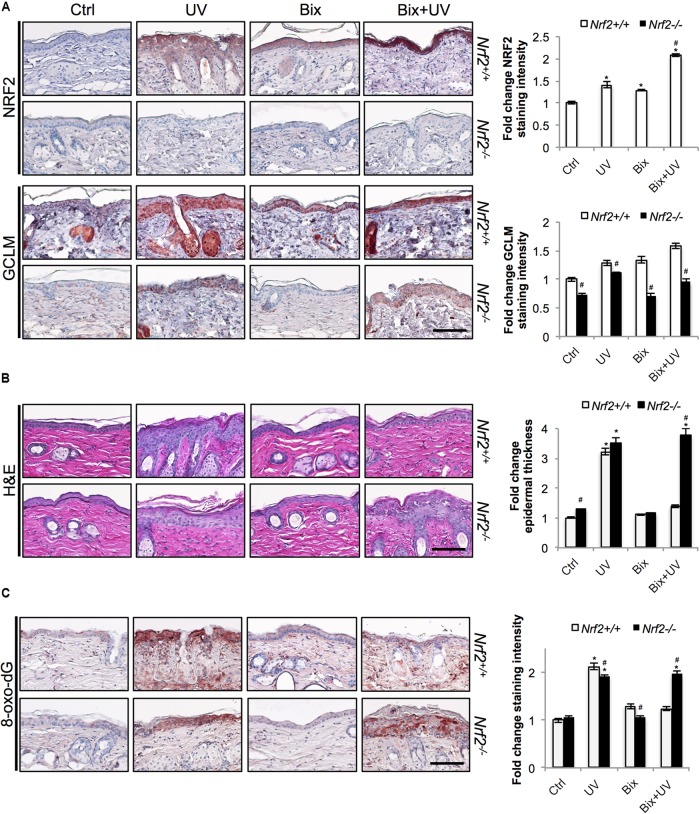
Topical bixin protects SKH1 mouse skin against acute photodamage in an NRF2-dependent manner. Histological analyses were performed on skin tissue sections collected from the SKH1 *Nrf2^+/+^* and *Nrf2^-/-^* mice from the study depicted on scheme 1 (**Figure 2A**). **(A)** IHC staining of NRF2 and GCLM epidermal expression. **(B)** H&E staining. **(C)** Staining of the oxidative DNA damage marker 8-oxo-deoxyguanosine (8-oxo-dG). Representative images are shown. Scale bar: 10 μm. Staining intensity was quantified, normalized, and represented as fold change compared to controls. *p* < 0.05: ^∗^compared to Ctrl, ^#^compared to *Nrf2^+/+^*.

Next, the protective effect of bixin-mediated NRF2 upregulation was tested in an acute skin photodamage *in vivo* model comparing *Nrf2^+/+^* and *Nrf2^-/-^* SKH1 hairless mice in order to substantiate the involvement of NRF2 in photoprotection. Since a single topical application of bixin caused only moderate NRF2 upregulation (**Figure 1**), two consecutive applications (24 h each) for enhanced NRF2 upregulation were employed, as specified in scheme 1 (**Figure 2A**; “2-day topical bixin regimen”). Twenty-four hours after the second topical application, mice were irradiated with UV and skin tissues were analyzed a day later for effects on acute photodamage. As expected, topical bixin induced the NRF2 pathway, as evidenced by immunoblot detection of increased expression of NRF2 (6.8-fold induction) and its target genes (TRXR1, NQO1, HO1, GCLM, OGG1, p62; fourfold maximum induction) in *Nrf2^+/+^* mice (**Figure 2B**). Likewise, UV treatment caused upregulation of NRF2 (twofold), consistent with earlier observations in SKH1 mice (Tao et al., 2015). Similar results were obtained using immunohistochemical analyses indicating increased epidermal staining for NRF2 and GCLM observable in response to UV, bixin, and bixin + UV treatment (**Figure 3A**). In contrast, modulation of NRF2 target gene expression in *Nrf2^-/-^* mice was much attenuated, a finding consistent with both the complete absence of NRF2 and the partial involvement of other stress response pathways regulating expression of general stress response proteins, such as HO1 (2.8-fold induction; **Figure 2B**).

Next we determined that topical bixin confers NRF2-dependent protection against UV-induced epidermal hyperproliferation and oxidative DNA damage. Histological analysis employing H&E staining confirmed that UV irradiation caused epidermal thickening observable in both *Nrf2^+/+^* (3.2-fold) and *Nrf2^-/-^* (3.5-fold) mice (**Figure 3B**). Strikingly, in bixin pre-treated *Nrf2^+/+^* mice (1.4-fold), epidermal thickening was greatly reduced, an effect absent from *Nrf2^-/-^* mice (3.8-fold). Importantly, UV exposure also increased epidermal occurrence of the oxidative DNA lesion 8-oxo-dG, detected by immunohistochemical analysis (**Figure 3C**). Again, occurrence of 8-oxo-dG lesions was greatly diminished by bixin pre-treatment in *Nrf2^+/+^* mice only (1.2-fold; **Figure 3C**), a protective effect consistent with the upregulation of the NRF2 target gene OGG1, encoding an 8-oxo-dG-directed repair glycosylase (**Figure 2B**). In contrast, in *Nrf2^-/-^* mice, UV-induced 8-oxo-dG lesions were extensive and persisted irrespective of bixin treatment (1.9-fold for UV and Bix + UV).

### Topical Bixin Activates NRF2 in C57BL/6J Mouse Skin Exposed to PUVA (Psoralen + UVA) Photodamage

**FIGURE 4:**
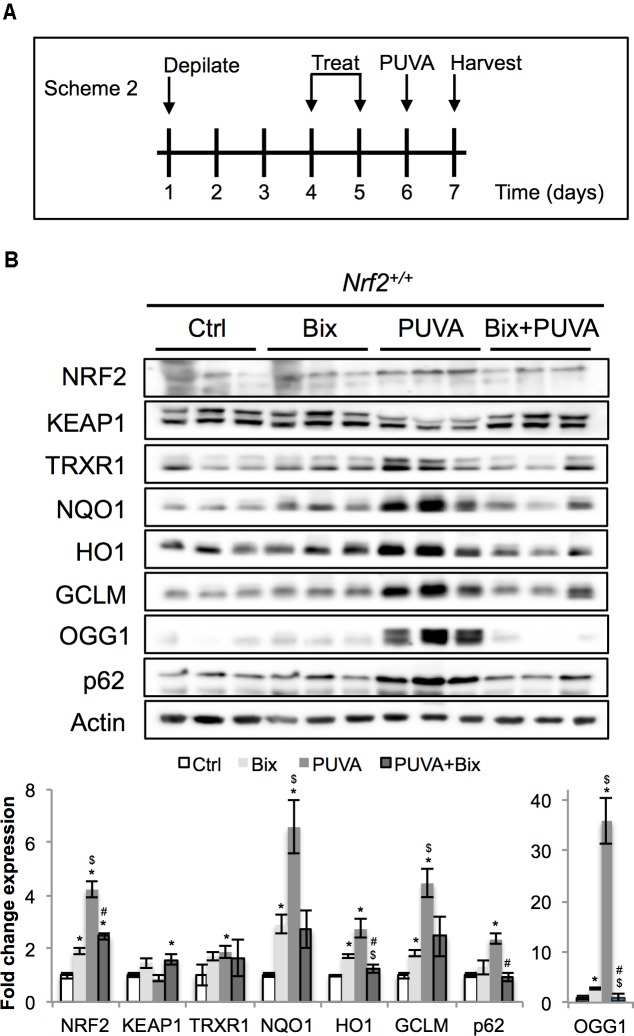
Topical bixin activates NRF2 and prevents acute PUVA-induced damage in C57BL/6J mouse skin. **(A)** Treatment scheme for acute PUVA study in C57BL/6J *Nrf2^+/+^* mice. **(B)** Protein expression of NRF2 and its downstream genes in skin tissue lysates as assessed by immunoblot analyses. Protein expression was quantified, normalized, and represented as fold change compared to controls. Blots of three representative samples for each group are shown. *p* < 0.05: ^∗^compared to Ctrl, ^#^compared to PUVA, ^$^compared to Bix.

Recent evidence indicates a role of NRF2 in protection of hair follicles and melanocytes against oxidative stress (Marrot et al., 2008; Jadkauskaite et al., 2017). Therefore, after demonstrating skin photoprotection by topical bixin in SKH1 mice, we also explored the feasibility of bixin-dependent suppression of stress-induced loss of hair pigmentation in C57BL/6J *Nrf2^+/+^* versus *Nrf2^-/-^* mice (**Figure 4**). To this end, we utilized an established mouse model employing topical PUVA (psoralen + UVA) for phototherapy-induced hair graying that occurs downstream of skin oxidative stress (Emerit et al., 2004; Anbar et al., 2012).

Before initiating the animal model, we examined bixin effects on human primary epidermal melanocytes (Supplementary Figure S1), experiments performed in analogy to our previously published studies on primary human keratinocytes (Tao et al., 2015). To this end, HEMnLP were treated with bixin followed by analyses of NRF2 activation. A robust induction of NRF2 protein levels was observed at 4 h (1.8-fold for 20 μM and 2.5-fold for 40 μM), and NRF2 levels went back to basal values at 16 h (Supplementary Figure S1A, top panel), consistent with the notion that bixin is a canonical NRF2 activator. Consistently, increased expression of NRF2 target genes (TRXR1, NQO1, p62, HO1; average twofold increase, sixfold for HO1) was detected by immunoblot analysis (Supplementary Figure S1A, bottom panel). Moreover, bixin pre-treatment protected melanocytes against hydrogen peroxide (H_2_O_2_)-induced loss of viability, observable in a chronic exposure model of oxidative stress (Supplementary Figure S1B). These results suggest that bixin effectively activates NRF2 in primary melanocytes and that bixin treatment can protect against oxidative stress-induced loss of viability.

Next, following our pilot experiment in SKH1 mice, we analyzed the acute effects of topical bixin used with or without PUVA on C57BL/6J *Nrf2^+/+^* mouse skin. As indicated in scheme 2 (**Figure 4A**), mice were depilated and skin was allowed to recover for 2 days before topical application of vehicle control or 1% bixin. One day after the second topical treatment, mouse skin received topical 8-MOP for 30 min, followed by UVA exposure. One day after PUVA, mouse skin was harvested and subjected to immunoblot analysis, demonstrating that topical bixin induces a twofold increase in cutaneous NRF2 and expression of NRF2 target genes (TRXR1, p62, NQO1, HO1, GCLM, OGG1) in C57BL/6J *Nrf2^+/+^* mice (**Figure 4B**), an effect similar to what had been observed in SKH1 mice (**Figures 1**, **2**). Interestingly, PUVA treatment caused pronounced NRF2 activation, an effect attenuated by bixin pre-treatment (>4-fold for PUVA versus twofold for Bix + PUVA).

### Topical Bixin Confers Protection Against PUVA-Induced Hair Graying in *Nrf2^+/+^* But Not in *Nrf2^-/-^* C57BL/6J Mice

**FIGURE 5:**
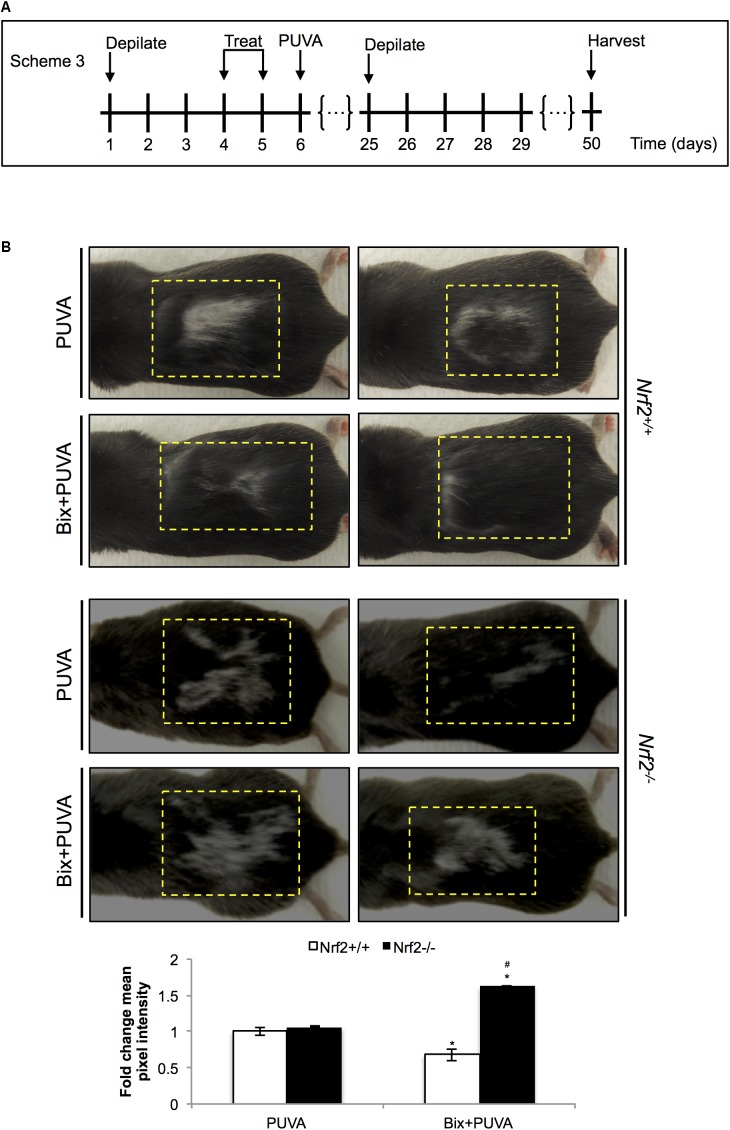
Topical bixin prevents PUVA-induced hair graying in C57BL/6J mouse skin. **(A)** Treatment scheme for PUVA-induced hair graying study in C57BL/6J *Nrf2^+/+^* and *Nrf2^-/-^* mice. **(B)** Representative images of PUVA-induced hair graying. The yellow lines delineate the area originally depilated and treated. Mean pixel intensity (whiteness) was calculated per treatment area, normalized, and expressed as fold change to *Nrf2^+/+^* PUVA values. *p* < 0.05: ^∗^compared to *Nrf2^+/+^* PUVA, ^#^compared to *Nrf2^+/+^* Bix + PUVA.

After substantiating NRF2 activation in C57BL/6J mouse skin, we tested the ability of topical bixin to suppress PUVA-induced hair graying as a function of NRF2 expression. To this end, we performed an experiment as described in scheme 3 (**Figure 5A**), in which C57BL/6J *Nrf2^+/+^* and *Nrf2^-/-^* mice were first depilated, followed by topical bixin and subsequent PUVA exposure. After one more cycle of depilation and hair regrowth over a period of 50 days, hair color was inspected in the back skin area that had undergone PUVA exposure with or without bixin pre-treatment. PUVA exposure caused pronounced hair graying in both *Nrf2^+/+^* and *Nrf2^-/-^* C57BL/6J mice (**Figure 5B**), an effect on hair pigmentation consistent with the published literature (Emerit et al., 2004). Strikingly, topical bixin pre-treatment greatly prevented the occurrence of this photodamage-induced phenotype, an effect observed only in *Nrf2^+/+^* mice (**Figure 5B**). Paradoxically, bixin pre-treated *Nrf2^-/-^* C57BL/6J mice displayed an increased occurrence of PUVA-induced gray hair (1.6-fold), an NRF2-independent effect of this pleiotropic agent that remains to be explored mechanistically (Rojo de la Vega et al., 2017). Taken together, these results indicate that upregulation of cutaneous NRF2 using topical bixin can antagonize phototherapy-induced hair graying in C57BL/6J mice.

## Discussion

Sunscreen-based photoprotection is an effective strategy to reduce skin exposure to solar UV light, and the development of more effective or synergistic molecular strategies acting through mechanisms different from photon absorption has recently attracted much attention (Rojo de la Vega et al., 2017). Cumulative evidence demonstrates that activation of the NRF2 pathway is essential for the maintenance of skin integrity and function in response to solar UV and other environmental stressors (Schafer and Werner, 2015; Jadkauskaite et al., 2017; Rojo de la Vega et al., 2017). Importantly, topical application of NRF2 inducers, e.g., the synthetic NRF2-activator TBE-31, has shown pronounced photoprotective and photochemopreventive activity in murine skin, and suppression of solar UV-induced human skin erythema was achieved by topical application of a standardized broccoli extract delivering the NRF2 inducer sulforaphane (Kalra et al., 2012; Knatko et al., 2015; Rojo de la Vega et al., 2017).

Here, we provide *in vivo* evidence demonstrating for the first time that topical application of bixin can protect skin against UV-induced acute photodamage and PUVA-induced loss of hair pigmentation in an NRF2-dependent manner in SKH1 and C57BL/6J mice, respectively. First, we demonstrated that a topical bixin formulation activates the NRF2 pathway in SKH1 mouse skin (**Figure 1**). Next, we demonstrated that topical bixin protects skin against UV-induced oxidative stress and epidermal hyperproliferation by modulation of the expression of NRF2 target genes (**Figures 2**, **3**), in agreement with our previous observations that systemic bixin administration activates NRF2 signaling in epidermal keratinocytes and protects mouse skin against acute UV photodamage and oxidative DNA lesions (Tao et al., 2015). Bixin-dependent NRF2 activation could protect from UV-induced damage by detoxifying ROS via upregulation of glutathione synthesis (GCLM) and antioxidant enzymatic systems (TRXR1, NQO1, HO1), promoting clearance of oxidized or damaged proteins or lipids through autophagy activation (p62), and promoting DNA repair (OGG1). Even though bixin has been reported to enhance skin barrier function by activation of PPARα/γ signaling and to inhibit TLR4/NFκB signaling, the *in vivo* experiments presented here indicate that the photoprotective effects achieved by topical bixin application depend largely on NRF2 activation, since the protection is lost in *Nrf2^-/-^* mice.

To test bixin-dependent NRF2 protective effects on hair graying *in vivo*, we adapted an animal model of oxidative stress-induced hair graying employing PUVA in C57BL/6J mice (Emerit et al., 2004). First, we observed that bixin induces the NRF2 pathway in primary human melanocytes and preserves viability upon oxidative stress insults (Supplementary Figure S1). Next, it was observed that topical bixin activates NRF2 in C57BL/6J *Nrf2^+/+^* mice (**Figure 4**). Furthermore, topical bixin confers protection against PUVA-induced hair graying (**Figure 5**), an NRF2-dependent photoprotective effect not observed in *Nrf2^-/-^* mice. Previous research has shown that PUVA-induced hair graying can be antagonized by topical pre-treatment with superoxide dismutase (Emerit et al., 2004), and direct-acting antioxidants protect dermal fibroblasts from PUVA-induced oxidative stress and premature senescence (Briganti et al., 2008). It is therefore feasible that bixin protection against PUVA-induced hair graying is a result of NRF2-dependent modulation of antioxidant responses that might also involve cytoprotective paracrine interactions between keratinocytes and melanocytes, as recently described (Arck et al., 2006; Jadkauskaite et al., 2017; Jeayeng et al., 2017). However, the specific mechanisms and molecular effectors (including cytokines) involved in NRF2-dependent protection against phototherapy-induced hair graying remain to be determined. Various NRF2 controlled stress response pathways may be involved in melanocyte protection against environmental electrophilic impact and stress-induced hair graying, to be elucidated in mechanistic follow-up studies (Arck et al., 2006; Jadkauskaite et al., 2017; Janjetovic et al., 2017; Jeayeng et al., 2017). It should also be mentioned that melatonin and its metabolites protect melanocytes against UVB-induced damage through NRF2-mediated pathways, a cytoprotective mechanism that might suppress skin environmental stress (Janjetovic et al., 2017; Slominski et al., 2017, 2018). Apart from upregulation of cytoprotective antioxidant and repair pathways in the cellular components of the hair follicle pigmentary unit, NRF2 regulation of hair follicle stem cells and improved cellular regeneration and wound healing may also contribute to maintenance of hair pigmentation under stress conditions (Panich et al., 2016; Jadkauskaite et al., 2017).

Based on the favorable toxicity profile of this FDA-approved apocarotenoid used worldwide as a food additive and topical cosmetic ingredient devoid of provitamin A activity, cutaneous NRF2 activation by bixin may have therapeutic potential for pharmacological protection of skin barrier function against environmental insults. Ethno-pharmacological use of topical bixin preparations is well documented both for cosmetic and therapeutic indications as recently reviewed (Rojo de la Vega et al., 2017). Indeed, NRF2-dependent pharmacological improvement of diabetic wound healing has been demonstrated (Long et al., 2016). Moreover, a potential therapeutic benefit of topical bixin might also be relevant to NRF2-dependent amelioration of psoriatic impairment of skin barrier function as well as radiation-induced dermatitis, based on cutaneous modulation of NRF2 in the absence of systemic changes that would protect tumor tissue against radiotherapy (Reisman et al., 2014; Nakagami and Masuda, 2016; Helwa et al., 2017; Jeong et al., 2017). Future experiments will examine if topical NRF2 activation by bixin may represent a novel molecular strategy for human skin photoprotection, potentially complementing conventional sunscreen-based approaches.

## Author Contributions

MR performed the experiments and analyzed the data. MR and GW designed the experiments. GW and DZ supervised the project. MR, GW, and DZ wrote the manuscript.

## Conflict of Interest Statement

The authors declare that the research was conducted in the absence of any commercial or financial relationships that could be construed as a potential conflict of interest.

## Abbreviations

8-MOP: 8-methoxypsoralen
8-oxo-dG: 8-oxo-deoxyguanosine
GCLM: glutamate cysteine ligase, modifier subunit
HEMnLP: human epidermal melanocytes, neonatal, low pigmented
HO-1: hemeoxygenase 1
KEAP1: kelch ECH-associated protein 1
NRF2: nuclear factor erythroid 2 (E2)-related factor 2
OGG1: 8-oxoguanine DNA glycosylase
PEG400: polyethylene glycol 400
PUVA: psoralen + UVA
TRXR1: thioredoxin reductase 1
