# Supplementary figures and images for: Topical Bixin Confers NRF2-Dependent Protection Against Photodamage and Hair Graying in Mouse Skin
Source: Front Pharmacol. 2018 Mar 27;9:287. doi: 10.3389/fphar.2018.00287 (PMC5880955; doi:10.3389/fphar.2018.00287)

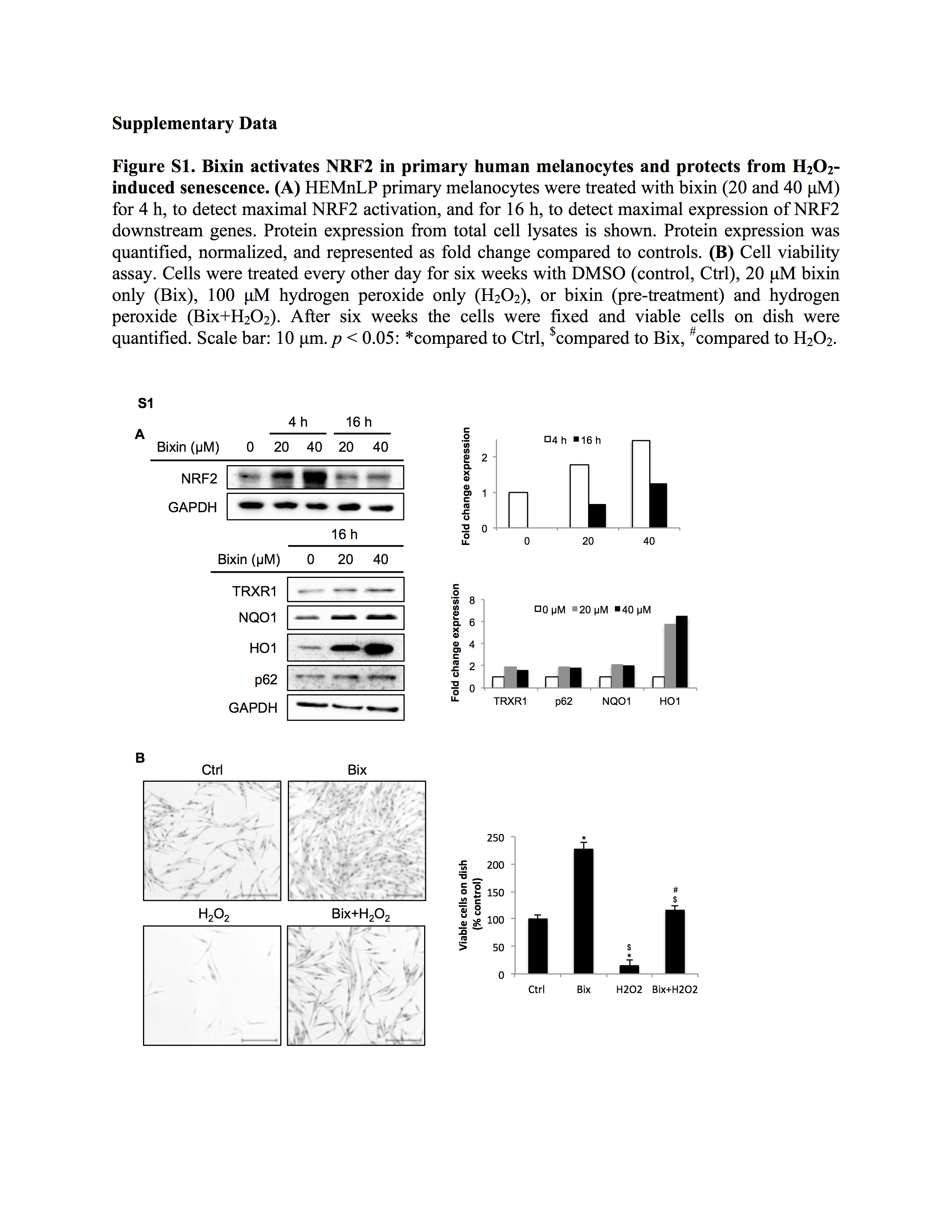

Supplement: Supplementary file 1 [file Image_1.tiff]
